# The Structure and Function of Acylglycerophosphate Acyltransferase 4/ Lysophosphatidic Acid Acyltransferase Delta (AGPAT4/LPAATδ)

**DOI:** 10.3389/fcell.2019.00147

**Published:** 2019-08-02

**Authors:** Mikhail A. Zhukovsky, Angela Filograna, Alberto Luini, Daniela Corda, Carmen Valente

**Affiliations:** Institute of Biochemistry and Cell Biology and Institute of Protein Biochemistry, National Research Council, Naples, Italy

**Keywords:** acyltransferase, AGPAT, LPAAT, phosphatidic acid, lysophosphatidic acid, Golgi complex, membrane fission, BARS

## Abstract

Lipid-modifying enzymes serve crucial roles in cellular processes such as signal transduction (producing lipid-derived second messengers), intracellular membrane transport (facilitating membrane remodeling needed for membrane fusion/fission), and protein clustering (organizing lipid domains as anchoring platforms). The lipid products crucial in these processes can derive from different metabolic pathways, thus it is essential to know the localization, substrate specificity, deriving products (and their function) of all lipid-modifying enzymes. Here we discuss an emerging family of these enzymes, the lysophosphatidic acid acyltransferases (LPAATs), also known as acylglycerophosphate acyltransferases (AGPATs), that produce phosphatidic acid (PA) having as substrates lysophosphatidic acid (LPA) and acyl-CoA. Eleven LPAAT/AGPAT enzymes have been identified in mice and humans based on sequence homologies, and their localization, specific substrates and functions explored. We focus on one member of the family, LPAATδ, a protein expressed mainly in brain and in muscle (though to a lesser extent in other tissues); while at the cellular level it is localized at the *trans-*Golgi network membranes and at the mitochondrial outer membranes. LPAATδ is a physiologically essential enzyme since mice knocked-out for *Lpaatδ* show severe dysfunctions including cognitive impairment, impaired force contractility and altered white adipose tissue. The LPAATδ physiological roles are related to the formation of its product PA. PA is a multifunctional lipid involved in cell signaling as well as in membrane remodeling. In particular, the LPAATδ-catalyzed conversion of LPA (inverted-cone-shaped lipid) to PA (cone-shaped lipid) is considered a mechanism of deformation of the bilayer that favors membrane fission. Indeed, LPAATδ is an essential component of the fission-inducing machinery driven by the protein BARS. In this process, a protein-tripartite complex (BARS/14-3-3γ/phosphoinositide kinase PI4KIIIβ) is recruited at the *trans*-Golgi network, at the sites where membrane fission is to occur; there, LPAATδ directly interacts with BARS and is activated by BARS. The resulting formation of PA is essential for membrane fission occurring at those spots. Also in mitochondria PA formation has been related to fusion/fission events. Since PA is formed by various enzymatic pathways in different cell compartments, the BARS-LPAATδ interaction indicates the relevance of lipid-modifying enzymes acting exactly where their products are needed (i.e., PA at the Golgi membranes).

## Introduction

Lysophosphatidic acid acyltransferases are an emerging family of enzymes that catalyze the production of PA using LPA and acyl-CoA (Korbes et al., 2016). The product of this enzymatic reaction, PA is involved in several essential cellular functions based on its unique properties as: (i) precursor for the biosynthesis of all glycerophospholipids and triacylglycerol (TAG); (ii) important membrane remodeling metabolite involved in the intracellular transport; and (iii) precursor of bioactive lipid mediators implicated in cell survival, proliferation, and tumor progression (Kume and Shimizu, 1997; Lu et al., 2005; Eto et al., 2014). The LPA is first synthetized, in the *de novo*-Kennedy pathway, via acylation of glycerol 3-phosphate (G3P) by the glycerol-3-phosphate acyltransferase (GPAT) enzymes that use acyl-CoAs as donors. Then, another fatty acid moiety (often an unsaturated one in eukaryotes) is incorporated at the *sn-*2 position on the LPA glycerol backbone to form PA by the 1-acylglycerol-3-phosphate acyltransferase enzyme (AGPAT, also known as LPA acyltransferase: LPAAT).

Eleven AGPAT enzymes, named AGPAT 1-11, have been identified in mice and humans based on the homology of their primary sequences (Kume and Shimizu, 1997). All AGPATs display highly conserved structural motif (Coleman and Mashek, 2011) and exhibit acyltransferase activity, using acyl-CoA and lysophospholipid as acyl-donor and acyl-acceptor, respectively (Kume and Shimizu, 1997). AGPAT enzymes have been named based on their substrate specificities and order in which their cloning have been reported. AGPATs 1, 2, 3, 4, and 5 specifically prefer LPA to form PA and, as such, are also known as LPAATα, β, γ, δ and ε, respectively, while AGPATs 6-11 are classified as lysophospholipid acyltransferases (LPLATs) or GPATs based on their substrate specificities (Yamashita et al., 2014).

Of note, recent studies indicate that each LPAAT enzyme is responsible for the production of a distinct and specific pool of PA required to affect downstream lipid metabolism that mediates specific cellular and organelle membrane lipid composition, which, in turn, controls physiological functions (Bradley and Duncan, 2018). Loss of function of a single LPAAT influences different downstream lipid biosynthetic pathways generating distinct pathophysiological consequences, including embryonic lethality, lipodystrophy, impaired spatial learning and memory (Bradley and Duncan, 2018). Moreover, LPAAT enzymes show differential subcellular localization together with a differential expression profiles within tissues or organs as well as preference toward specific fatty acyl-CoA donor moieties (Shindou et al., 2009). These aspects may explain the existence of different LPAAT isoforms in nature able to specifically modulate downstream glycerolipid pathways in different tissues and organs for maintenance of normal physiology. As a consequence, loss of specific LPAAT cannot be functionally and biochemically replaced by other LPAATs (Bradley and Duncan, 2018). Tissue distribution patterns reveal an ubiquitous expression of LPAATα and LPAATγ, while LPAATβ, δ, and ε display distinct tissue-specific profiles (Takeuchi and Reue, 2009).

Following a brief overview on phylogenetic tree of LPAAT genes and on the unique properties of PA, the product of the reaction catalyzed by these enzymes, we analyze the conserved acyltransferase motifs required for enzymatic activity with the spotlight on LPAATδ member. We first discuss the membrane topology of human LPAATδ based on the recently resolved structure of bacterial LPAAT. We then highlight the role of the specific LPAATδ-produced PA pool in lipid metabolism and tissue functions. Finally, we report on recent advances in our understanding of the membrane fission event involving LPAATδ and occurring at the *trans*-side of the Golgi complex. Specifically, we discuss how this enzyme assembles with other proteins in a protein complex and how this machinery is regulated and operates in the formation of the basolaterally directed post-Golgi carriers.

## LPAAT Family

The LPAAT enzymes are an ancient gene family that were first functionally described in 1956 (Kennedy and Weiss, 1956). It belongs to the subgroup 3 of the membrane bound *O*-acyltransferase (MBOAT) superfamily (Chang et al., 2011; Korbes et al., 2016). LPAAT genes were found in all three domains of life: Archaea, Bacteria and Eukarya where the LPAAT enzymes play key metabolic roles. At least one LPAAT gene was found in each of the eukaryotic genome examined (Korbes et al., 2016). Some eukaryotic species contain a few LPAAT genes (e.g., thirteen in the soya bean *Glycine max*), whereas yeast *Saccharomyces cerevisiae* possesses only one LPAAT gene, SLC1 (Korbes et al., 2016). Five LPAAT enzymes namely LPAATα-LPAATε are present in mammals (Korbes et al., 2016; Bradley and Duncan, 2018).

The phylogenetic tree analyses of LPAAT genes from prokaryotic to eukaryotic species based on phosphate acyltransferase (PlsC) domain identified three distinct clusters designed as cluster I, II, and III (Korbes et al., 2016). Cluster I is the most ancient and contains plant LPAATα and LPAATβ, prokaryotic and fungal LPAATs, as well as animal LPAATα and LPAATβ. Cluster II consists of animal AGPAT6, AGPAT10, AGPAT7, AGPAT9, and AGPAT11. Cluster III is divided into subclusters: IIIa contains animal LPAATγ, LPAATδ and plant LPAATβ, LPAATγ; IIIb is composed of plant LPAATδ, LPAATε, fungal LPAAT and animal AGPAT8; and IIIc includes animal LPAATε. Of note, two different proteins LPAATγ and LPAATδ in animals appeared due to a duplication event (Korbes et al., 2016). The sequence of human LPAATδ is more similar to the sequence of human LPAATγ (ca. 60%) than to that of other three human LPAATs, see Supplementary Tables S1, S3. A few residues are typical of the members of subcluster IIIa: they are highly conserved in LPAATγ and LPAATδ orthologs, but are rare among other animal LPAATs. A good example of such residues is tryptophan (W, at position 106 in human LPAATδ), five residues downstream of aspartic acid (D) belonging to the conserved NHxxxxD sequence, see Supplementary Tables S1–S4.

Lysophosphatidic acid acyltransferase γ, as LPAATα, is ubiquitously expressed with high mRNA levels in adipose tissue, liver and heart (Yuki et al., 2009). LPAATγ, in addition of being localized, like LPAATα and LPAATβ, at the ER (Agarwal et al., 2011; Yamashita et al., 2014), is a Golgi-resident enzyme that controls Golgi structure and retrograde transport from the Golgi complex to the ER. Depletion of LPAATγ causes Golgi membrane fragmentation and severe impairment of COPI-coated vesicle formation (Schmidt and Brown, 2009; Yang et al., 2011). Indeed, inhibition of LPAATγ enzyme impairs the release of COPI buds as vesicles from the Golgi complex. This is due to the role of LPAATγ activity in controlling the membrane fission of these buds from the Golgi membranes (Yang et al., 2011). Of note, LPAATγ prefers C20:4 and C22:6 fatty acyl-CoA donors (Yuki et al., 2009; Koeberle et al., 2010, 2012) and this might explain the original evidence of acyl-CoA’s involvement in COPI-coated vesicle formation (Pfanner et al., 1989).

Here we selectively focus on LPAATδ because of its emerging role in membrane remodeling (fission and fusion) and related diseases caused by defective lipid metabolism and/or altered membrane composition, while for an extensive and accurate description of the other LPAAT enzymes the reader is referred to excellent reviews (Yamashita et al., 2014; Bradley and Duncan, 2018).

## Phosphatidic Acid: the Product of the LPAAT-Catalyzed Enzymatic Reaction

**FIGURE 1:**
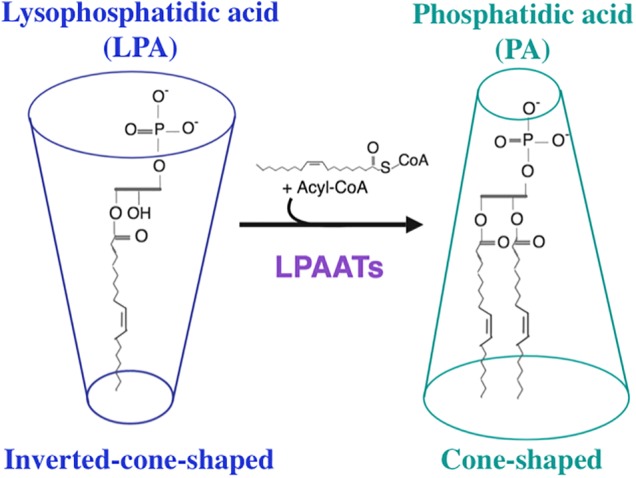
Enzymatic reaction catalyzed by lysophosphatidic acid acyltransferase enzymes. LPAATs promote the addition of an unsaturated fatty acid from Acyl-CoA (as acyl donor) to lysophosphatidic acid (LPA, as acyl acceptor) to form phosphatidic acid (PA). This reaction converts an inverted-cone-shaped lipid (LPA) into a cone-shaped lipid (PA).

Phosphatidic acid is composed of a three-carbon glycerol backbone, to which two fatty acyl chains are ester-linked at positions C-1 and C-2, and a phosphate is ester-linked at position C-3. On the basis of their shapes, lipids can be divided into three classes (Cullis and De Kruijff, 1979; Corda et al., 2002; Chernomordik and Kozlov, 2003; and references therein): cone-shaped lipids, inverted-cone-shaped lipids, and cylindrical lipids. Lipids whose tails are wider than headgroups are cone-shaped, lipids whose headgroups are wider than tails are inverted-cone-shaped, and lipids whose headgroups are approximately as wide as tails are cylindrical. PA is a cone-shaped lipid, whereas LPA possesses an inverted-cone shape (Kooijman et al., 2005b; Figure 1). Thus, the interconversion from LPA to PA mediated by LPAAT enzymes destabilizes the organization of the lipid bilayer causing a distortion of this bilayer, a process that supports membrane fission (Barr and Shorter, 2000; Shemesh et al., 2003; Pagliuso et al., 2016). Moreover, both PA and LPA are charged negatively (Kooijman et al., 2005a). Hence, PA possesses an unique combination of cone shape and negative charge (Tanguy et al., 2019). Such unique property of PA allows it to recruit various proteins to the membrane (Stace and Ktistakis, 2006). According to the electrostatic/hydrogen bond switch mechanism (Kooijman et al., 2007), the PA charge is able to change from -1 to -2 due to the deprotonation of PA headgroup, caused by formation of the hydrogen bond of positively charged amino acid residue (lysine or arginine) with this headgroup. This change can stabilize the protein-lipid interaction (Kooijman et al., 2007). Due to its phosphomonoester headgroup whose p*K_a_* value is within physiological range, PA acts as a pH biosensor (Young et al., 2010; Shin and Loewen, 2011). Eventhough the content of PA in the cellular membrane is usually low, ∼1–4% (Zinser et al., 1991; Young et al., 2010; Zegarlinska et al., 2018), this lipid is involved in many important biological processes such as induction of membrane curvature, membrane trafficking, recruitment of proteins to membranes, regulation of catalytic activity of various enzymes, signal transduction, cytoskeletal organization and regulation of gene expression (Kooijman and Burger, 2009; Raben and Barber, 2017; Kameoka et al., 2018; Zegarlinska et al., 2018; Tanguy et al., 2019; and references therein). Various phospholipids, such as PC, PE, PI and CL are synthesized through the common precursor, PA (Yamashita et al., 2014). In the Lands’ cycle, these phospholipids can be deacylated and reacylated at the *sn*-2 position in a coordinated and concerted control cycle by phospholipases A_2_ (PLA_2_s) and LPLATs actions, respectively (Bankaitis, 2009; Ha et al., 2012). This cycle is essential to generate the membrane asymmetry and diversity that support membrane fluidity and curvature required for fundamental biological functions. Thus, PA is involved, directly or indirectly, in the biosynthesis of most phospholipids (Kassas et al., 2017). Hence, also enzymes that catalyze the synthesis of PA play very important role in biological processes.

**FIGURE 2:**
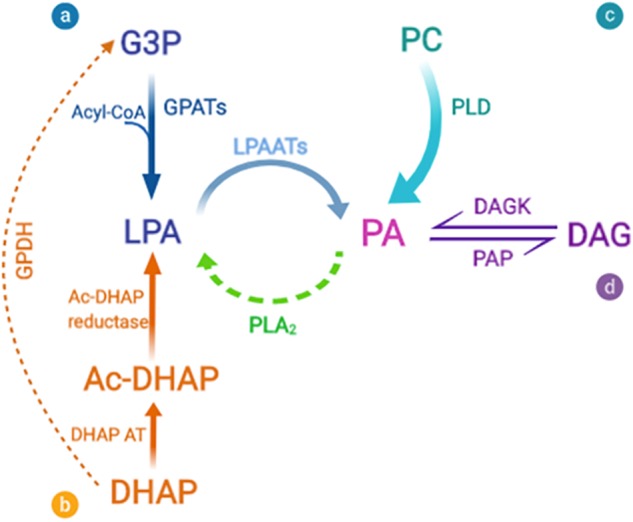
Biosynthetic pathways for phosphatidic acid production. Phosphatidic acid (PA) can be generated by three major routes: **(a-b)** The *de novo* synthesis via lysophosphatidic acid (LPA) formation that occurs via two different acylation pathways. **(a)** The first and main synthesis route is the glycerol 3-phosphate (G3P) pathway. G3P is acylated by a glycerol 3-phosphate acyltransferase (GPAT) to form LPA. **(b)** The second pathway of LPA formation involves acylation of dihydroxyacetone phosphate (DHAP) by DHAP acyltransferase (DHAP AT) via the 1-acylDHAP (Ac-DHAP) pathway followed by Ac-DHAP reductase-mediated reduction. LPA can then be further acylated by the addition of an unsaturated fatty acid (generally arachidonate) to form PA, via a lysophosphatidic acid acyltransferase (LPAAT). The inverse reaction is mediated by phospholipase A_2_ (PLA_2_), which thus converts PA into LPA. Both of these reactions are of particular significance for the geometry of the membrane bilayers, since cone-shaped PA is converted to inverted-cone-shaped LPA (and *vice* *versa*), thus facilitating rapid changes in membrane curvature. **(c)** PA is also formed by the breakdown of other phospholipids, and in particular by the activity of phosphatidylcholine (PC)-specific phospholipase D (PLD). **(d)** Finally, PA can be dephosphorylated by PA phosphatases (PAPs), to form diacylglycerol (DAG), a strongly conical component of the bilayer, which due to its small and uncharged headgroup, has spontaneous transbilayer movement (flip-flop). The opposite reaction is catalyzed by diacylglycerol kinases (DAGKs). PA and DAG have been shown to be in dynamic equilibrium, and this mechanism can affect the composition and curvature of both leaflets of the bilayer.

In all organisms, PA can be produced by one of three major routes (Foster et al., 2014; Vodicka et al., 2015; Kassas et al., 2017): *de novo* synthesis in which the final step is acylation of LPA by LPAAT enzymes, phosphorylation of DAG by DAGKs, and hydrolysis of phospholipids by PLD (Figure 2). LPAATs are members of the family of AGPAT enzymes that specifically use LPA as acyl acceptor (Yamashita et al., 2014).

## Acyltransferase Motifs in LPAATs

The LPAAT enzymes contain four catalytic motifs I-IV (Takeuchi and Reue, 2009; Rottig and Steinbuchel, 2013; Yamashita et al., 2014), and here we report the sequence alignment of LPAATs from sixty species of different biological kingdoms, see Supplementary Tables S1, S3.

Motifs I and III are the most conserved, whereas motif IV is the least conserved. Motifs I and III are suggested to be involved in the interaction with the acyl acceptor, whereas motif IV is suggested to interact with acyl donor (Yamashita et al., 2014; Robertson et al., 2017; and references therein).

Catalytic motif I contains a conserved NHxxxxD sequence. In this sequence, the residue following histidine (H) is usually hydrophilic, whereas the 5th residue upstream this H and the residue preceding aspartic acid (D) are almost always hydrophobic, see Supplementary Tables S1, S3. The H belonging to this sequence acts as a general base to abstract proton from the hydroxyl group which has to be acylated (Pagac et al., 2011; Yamashita et al., 2014; and references therein). The role of D belonging to the NHxxxxD sequence is to maintain the lone pair of electrons on the Nε2 nitrogen of catalytic H so as to abstract a proton (Robertson et al., 2017; and references therein). Among sixty LPAATs from different species presented in the Supplementary Tables S1, S3, the H belonging to the catalytic motif I is absolutely conserved, whereas the D is conserved almost absolutely (present in 59 out of 60 sequences).

Catalytic motif II contains a conserved arginine (R). The residue that precedes this R is always (or almost always) hydrophilic, whereas amino acid residue that precedes this hydrophilic residue is usually hydrophobic. In LPAATs from animals and fungi, this R often belongs to the (F/Y)xxR pair, see Supplementary Tables S1, S3.

Catalytic motif III contains a conserved EGTR sequence. In most LPAAT enzymes, although not in mammalian LPAATδ, amino acid residue that is two residues upstream of glutamic acid (E) belonging to the EGTR sequence is phenylalanine (F), see Supplementary Tables S1, S3. In PlsC, a LPAAT from bacterium *Thermotoga maritima*, the only LPAAT for which the crystal structure is available, conserved R belonging to this EGTR sequence is ideally positioned to bind the 3′-phosphate of LPA (Robertson et al., 2017). Among sixty LPAATs from different species presented in Supplementary Tables S1, S3, E and glycine (G) of the catalytic motif III are also almost absolutely conserved. They are present, respectively, in 58 and 59 of the 60 sequences shown in Supplementary Tables S1, S3.

Catalytic motif IV contains a conserved proline (P), see (Yamashita et al., 2014; Korbes et al., 2016) and Supplementary Tables S1, S3. In most LPAAT enzymes, two residues preceding this P are hydrophobic, see Supplementary Tables S1, S3. Sometimes the residue following this P also plays a role in catalysis (Yamashita et al., 2014).

In mammalian LPAATα, mutations of conserved residues belonging to the calatylic motifs lead to the strong inhibition of catalytic activity. Among these mutations are the following: mutation of H of the motif I to alanine (A), mutations of D of the motif I to E or asparagine (N), mutation of R of the motif II to A, mutation of E of the motif III to D or glutamine (Q), mutation of G of the motif III to leucine (L), mutation of R of the motif III to A or lysine (K) (Yamashita et al., 2007, 2014).

Naturally occurring mutations in human LPAATβ, the best studied and characterized of all mammalian LPAATs, are associated with type one Berardinelli-Seip CGL (Agarwal et al., 2002; Magre et al., 2003; Agarwal, 2012; Subauste et al., 2012) and Brunzell syndrome (Fu et al., 2004). Some of the mutations causing the CGL affect residues belonging to the catalytic motifs and/or were shown to result in partial or complete inhibition of LPAATβ enzymatic activity. Specifically: (i) mutation of E to K at position 172 (E172K) belonging to the conserved EGTR sequence, within the catalytic motif III (Magre et al., 2003; Haghighi et al., 2012); (ii) deletion mutation 140delF affects F that belongs to the conserved FxxR pair within the catalytic motif II (Haque et al., 2005); and (iii) mutation of serine (S) to N at position 100 (S100N) changes the sequence within the catalytic motif I (Cortes et al., 2009).

Finally, single mutations H96A, D101A, and E176A in mouse LPAATγ (Yuki et al., 2009) and quintuple mutation N95A/H96A/D101A/E176A/G177A in human LPAATγ (Schmidt and Brown, 2009) lead to complete inhibition of LPAAT activity. Residues N95, H96, and D101 belong to the catalytic motif I, whereas residues E176 and G177 belong to the catalytic motif III.

## Animal LPAATδ Enzyme

Lysophosphatidic acid acyltransferase δ is a member of the LPAAT family (Lu et al., 2005; and references therein) that exhibits catalytic activity with LPA, but not with most other major lysophospholipids, such as LPC, LPE, LPS, LPI, LPG, MLCL, DLCL or with glycerol 3-phosphate (G3P) (Eto et al., 2014; Bradley et al., 2015). The unsaturated acyl-CoAs C22:6, C20:4, C18:1 followed by C16:0 are the preferred substrates of this enzyme with *V_max_* = 23.2 ± 2.4 nmol/min/mg and *K_m_* = 42.9 ± 2.9 μM for 18:0-LPA (Eto et al., 2014) and *V_max_* = 38 ± 1 nmol/min/mg, *K_m_* = 29 ± 1 μM for 18:1-LPA (Pagliuso et al., 2016). The highest LPAATδ catalytic activity was observed at pH 7.4 using 18:1-LPA and 18:1-acyl-CoA, with a minor decrease when pH rose to 7.6 and a sharp decrease when pH was lowered to 7.2 (Bradley et al., 2015).

The human *Lpaat*δ gene is on chromosome 6. Both human *Lpaat*δ and mouse *Lpaat*δ possess seven introns and five exons while *Arabidopsis* and rice *Lpaat*δ possess two introns and three exons with similar size (Korbes et al., 2016). Mammalian LPAATδ (residue numbers are for the human ortholog, UniProt accession number Q9NRZ5) contains a few conserved residues belonging to four catalytic motifs, including N95, H96 and D101 of the motif I, F143 and R146 of the motif II, E176 and G177 of the motif III, P206 of the motif IV. The H96A mutant of mouse LPAATδ shows much lower catalytic activity as compared to the wild type enzyme (Eto et al., 2014), and H96V mutant of human LPAATδ is inactive (Pagliuso et al., 2016), demonstrating that H96 residue is essential for catalysis. Moreover, this H96V mutation in human LPAATδ leads to the inhibition of the fission step during post-Golgi carrier formation indicating that LPAATδ catalytic activity is required for this process (Pagliuso et al., 2016). Based on the published results of the mutagenesis experiments with LPAATα (Yamashita et al., 2007, 2014) and LPAATγ (Yuki et al., 2009), we expect that the following mutations will also inhibit the catalytic activity of LPAATδ: D101A, D101E, D101N, R146A, E176A, E176D, E176Q, G177L, R179A, and R179K.

A crystal structure of PlsC, a LPAAT from bacterium *Thermotoga maritima* (*T. maritima*), was reported with the studies of the functional role of various PlsC residues (Robertson et al., 2017). Based on comparison among TmPlsC and other LPAATs, we can derive some conclusions concerning the structure of mammalian LPAATδ.

In TmPlsC, the phosphate group of LPA was suggested to interact with the highly conserved residues R159 (within catalytic motif III) and K105 (Robertson et al., 2017). This K105 is located between the catalytic motif I and a highly conserved P (P112 in TmPlsC). This K is highly conserved across all biological kingdoms except Archaea, see Supplementary Tables S1, S3. In human LPAATδ, K123 followed by K124 correspond to K105 from TmPlsC, see Supplementary Tables S1, S3. Based on this sequence alignment, we can conclude that in LPAATδ, K123 or, perhaps, K124 interact with the phosphate group of LPA. In the orthologs of animal LPAATδ, K corresponding to K123 of human LPAATδ is conserved, see Supplementary Tables S2, S4. We expect that in human LPAATδ, the catalytic activity of the K123A/K124A double mutant will be negligible.

## Membrane Topology of Human LPAATδ

The TmPlsC crystal structure is consistent with the organization in two domains: the N-terminal two-helix motif and the αβ-domain that contains all four catalytic motifs (Robertson et al., 2017). This molecule does not contain any TMDs, but its α1 helix (belonging to the N-terminal two-helix motif) enters and exits on the same side of the membrane, due to the presence of the G^25^G^26^ kink within this helix.

In Supplementary Tables S1, S3 the sequence alignment of TmPlsC, human LPAATδ and many other LPAATs from different biological kingdoms is indicated. One of TmPlsC aromatic residues suggested to interact with the apolar interior of the lipid bilayer, tryptophan W116 (Robertson et al., 2017) is highly conserved, see Supplementary Tables S1, S3. This W is located between catalytic motifs I and II and, more precisely, between highly conserved P (P112 in TmPlsC) and catalytic motif II. The W in this location is present in the LPAAT enzymes from many species. In human LPAATδ, W134 and W136 are located between highly conserved P (P130 in LPAATδ, part of highly conserved PxxG motif) and catalytic motif II. In LPAATδ, tryptophans at these positions are highly conserved throughout evolution, see Supplementary Tables S2, S4. We hypothesize that W134 and W136 from LPAATδ, like W116 from TmPlsC, interact with the apolar interior of the lipid bilayer.

The membrane topology of a few LPAATs, such as human LPAATα (Yamashita et al., 2007), human LPAATγ (Schmidt et al., 2010), *Saccharomyces cerevisiae* SLC1 (Pagac et al., 2011) and peanut LPAT4 (Chen et al., 2012), was studied experimentally as well as using bioinformatics. All enzymes reported in these studies are localized to the ER or Golgi membranes and contain a TMD between catalytic motifs I and II, while no TMD is foreseen between catalytic motifs II and III (see Figure 3 of Yamashita et al., 2014). This topological organization is quite unusual (motif I on one side of the membrane, and motifs II and III on the other side) and it can be explained by the need to bring in close proximity catalytic motifs I–IV that may penetrate into the membrane from the cytosolic or luminal side to act in concert (Yamashita et al., 2007, 2014; Chen et al., 2012).

Eto et al. (2014) predicted six TMDs in LPAATδ by HMMTOP transmembrane topology prediction server (Tusnady and Simon, 1998, 2001).

We would like to point out that no long hydrophobic stretches between catalytic motifs II and III and between catalytic motifs III and IV are present in LPAATδ (see Supplementary Tables S2, S4). Accordingly, it is reasonable to propose that no TMD characterizes the organization of this LPAATδ segment, and, hence, the three catalytic motifs II, III, and IV are located on the same side of the membrane. It should be noted, however, that the stretch L^126^AYVPIIGWMWYF^138^ between catalytic motifs I and II of human LPAATδ is very hydrophobic and that similar hydrophobic stretches are present in this location in LPAATδ orthologs from other animal species (Supplementary Tables S2, S4). It may be hypothesized that this stretch is a TMD and, hence, in LPAATδ, as in all four eukaryotic LPAATs mentioned above, a TMD is present between catalytic motifs I and II. However, this stretch (13 residues) is unusually short, and the vast majority of TMDs are longer (Singh and Mittal, 2016). As a consequence, if this stretch is indeed a TMD, the location of catalytic motifs I and II of LPAATδ on the different sides of the membrane would not favor catalysis (Yamashita et al., 2007; Schmidt et al., 2010; Pagac et al., 2011; Chen et al., 2012).

**FIGURE 3:**
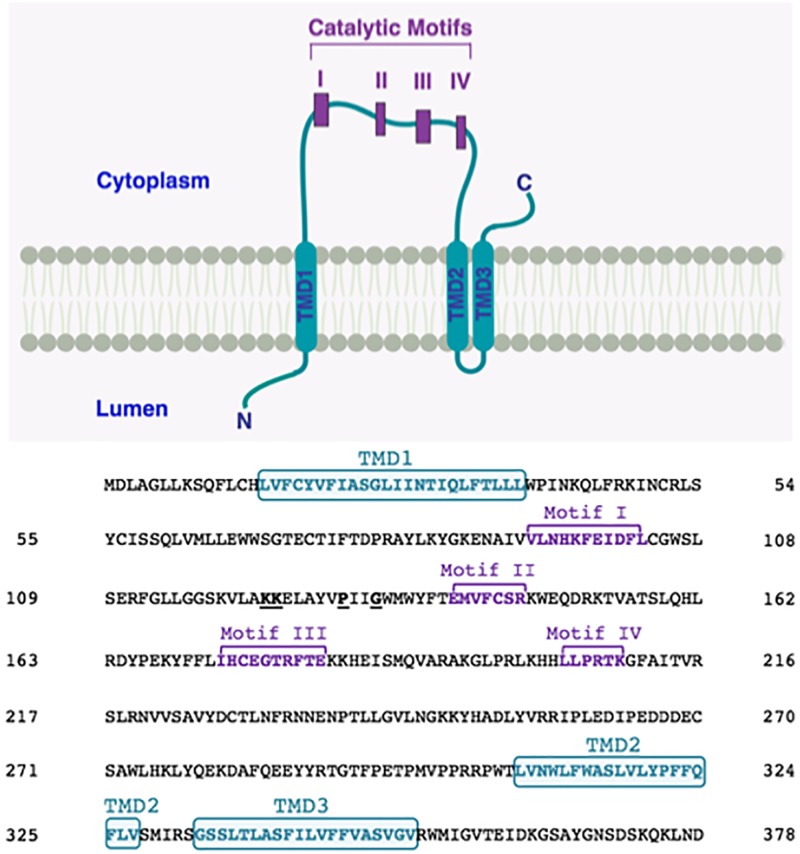
Proposed membrane topology of human LPAATδ. The transmembrane domains (TMD; in turquoise) were predicted by CCTOP prediction server (http://cctop.enzim.ttk.mta.hu) (Dobson et al., 2015) and indicated as TMD1 (amino acids 15-38), TMD2 (amino acids 308-327), and TMD3 (amino acids 333-352). The four Catalytic Motifs of LPAATδ are indicated as I-IV in purple, and their amino acid sequences are reported in the sequence alignment as Motif I (amino acids 93-103), Motif II (amino acids 140-146), Motif III (amino acids 173-182), and Motif IV (amino acids 204-209). Highly conserved proline P130 and glycine G133 in the PxxG motif between catalytic motifs I and II are highlighted. Lysines K123 and K124 are highlighted. The N-terminus of the protein is predicted to be located in the lumen while the C-terminus is predicted to be located in the cytoplasm (as indicated).

In order to bring some clarity, we decided to use CCTOP server^1^, a web-based application providing prediction of membrane protein topology. This server utilizes ten different state-of-the-art topology prediction methods, including HMMTOP (Dobson et al., 2015). We studied membrane topology of human LPAATδ using CCTOP. We found that, according to this prediction, with reliability 81.1, human LPAATδ contains only three TMDs: one TMD is upstream of the catalytic motif I, whereas two more TMDs are downstream of the catalytic motif IV. Hence, according to this prediction, all four catalytic motifs are located on the same side of the membrane (Figure 3).

We hypothesize that human LPAATδ, like TmPlsC, does not contain any TMDs between catalytic motifs I and II, and all four catalytic motifs are located on the same side of the membrane (Figure 3). Moreover, we hypothesize that in human LPAATδ, the L^126^AYVPIIGWMWYF^138^ stretch forms an α-helix, and this helix, like the α1 helix in TmPlsC, enters and exits on the same side of the membrane. In the α1 helix in TmPlsC, such topology is facilitated by the presence of the G^25^G^26^ kink in the middle of this helix. We believe that in human LPAATδ, the motif P^130^xxG^133^ plays a role of such kink. This PxxG motif is highly conserved among various LPAATs, see Supplementary Tables S1, S3, and is conserved across animal LPAATδ orthologs, see Supplementary Tables S2, S4. In proteins, kinks often coincide with the hinges (flexible regions that decouple pre-hinge and post-hinge portions of protein segment) (Cordes et al., 2002; Bright and Sansom, 2003; Hall et al., 2009). Molecular hinges often contain P residues (Sansom and Weinstein, 2000; Cordes et al., 2002; Bright and Sansom, 2003; Hall et al., 2009). The flexibility introduced by a P residue can be increased in the presence of G (Sansom and Weinstein, 2000; Cordes et al., 2002; Bright and Sansom, 2003) close to P, usually not more than four residues between P and G (Bright and Sansom, 2003).

The function of such hypothetical conformation of the L^126^AYVPIIGWMWYF^138^ stretch (entering and exiting on the same side of the membrane) might be, as suggested to the two-helix motif of TmPlsC (Robertson et al., 2017), to be a “fishing bobber” that suspends the LPAATδ active site close to the lipid molecules involved in reaction. Similar function of the short hydrophobic stretch between catalytic motifs I and II can be hypothesized also in other LPAATs.

## Physiological Functions of LPAATδ Enzyme in Mouse Model

Lysophosphatidic acid acyltransferase δ is most highly expressed in brain (Eto et al., 2014; Bradley et al., 2015, 2016, 2017), but also in muscle (Bradley and Duncan, 2018) and, to a lesser extent, in other tissues, such as lungs, intestines, epidermis, and spleen. At the level of the mouse central nervous system, LPAATδ is abundant at the brain stem, cortex, hippocampus, cerebellum and olfactory bulbs (Bradley et al., 2015).

The characterization of the physiological functions of LPAATδ is based on the analysis of the dysfunctions associated with *Lpaatδ* gene knockout in mouse model. The absence of this enzyme results in a wide range of alterations that include: cognitive impairment, impaired force contractility and altered visceral white adipose tissue depots.

The level of PI, PE, and PC is significantly lower (by 52, 32, and 38%, respectively) in the brain of *Lpaatδ^-^* mice (Bradley et al., 2015). However, the loss of *LPAATδ* did not influence the level of brain PA (Bradley et al., 2015) and this can be explained by redundancies in the LPAAT family. Indeed, an up-regulation of LPAATε, LPAATα and, even more, of LPAATβ has been found in the brain of *LPAATδ^-^* mice indicating a degree of compensation in PA synthesis (Bradley et al., 2015). Of note, this adaptation in PA brain content was not able to compensate the reduced levels of the other phospholipid species (PI, PC, and PE) observed in the brain of *Lpaatδ^-^* mice. This implies that the LPAATδ enzyme produces a specific pool of PA that is used as precursor to support the biosynthesis of PI, PC, and PE. The levels of the other major brain phospholipids were not affected indicating an unique function of LPAATδ in regulating the PI, PC and PE as downstream Kennedy pathway derivatives (Bradley et al., 2015).

*Lpaatδ^-^* mice have significant impairments in spatial learning and memory (Bradley et al., 2017). This phenomenon can be partly attributed to the drastically lower brain content of the NMDA receptor subunits (namely NR1, NR2A, and NR2B), and of the GluR1 subunit of the AMPA receptor. NMDA receptor and AMPA receptor are two glutamate-gated transmembrane proteins involved in synaptic plasticity and memory (Bradley et al., 2017). Such a significantly reduced neural content of these subunits, in turn, might be explained by the noticeable decrease in PI, PE, and PC in the brain of *Lpaatδ^-^* mice. The presence of these glycerophospholipids might be required to preserve the biophysical properties of neuronal membranes in order to generate the conditions for the correct assembly and function of these receptors (Bradley et al., 2017). Specific lipids have been reported to have a modulatory role in the structure and function of many membrane proteins (Opekarova and Tanner, 2003; Lee, 2004; Bogdanov et al., 2008; Zhukovsky et al., 2013; and references therein). We expect that phospholipids whose synthesis depends (directly or indirectly) on LPAATδ are required for the native conformation and normal function of a few other membrane proteins, and the absence of LPAATδ might lead to the disruption of the function of these proteins.

Lysophosphatidic acid acyltransferase δ possesses catalytic activity for DHA-CoA (Eto et al., 2014). DHA is required for neurite outgrowth in hippocampal neurons (Calderon and Kim, 2004), and its reduced levels is associated with the development of cognitive and neurodegenerative disorders. Hence, the high level of LPAATδ expression in the brain is consistent with DHA being abundant among brain phospholipids (Yamashita et al., 2014), suggesting an important role of LPAATδ in maintaining DHA in neural membranes (Eto et al., 2014).

Lysophosphatidic acid acyltransferase δ was found in various muscle types. However, it was detected at highest levels in soleus, a red oxidative fiber-type that is rich in mitochondria (Bradley and Duncan, 2018). This is consistent with the localization of LPAATδ to the outer mitochondrial membrane (Bradley et al., 2015). *Lpaatδ^-^* mice showed increased PA and PE contents on fiber-type composition that in turn is suggested to impair the force contractility in soleus. These effects seem not associated to LPAATδ-related mitochondria dysfunction; as such, the *Lpaatδ^-^* mice did not exhibit impaired mitochondrial function or reduced mitochondrial content (Bradley et al., 2015). In Bradley et al. (2018), a compensatory mechanism in PA synthesis was also reported. Indeed, LPAATβ and LPAATε are specifically upregulated in soleus of *Lpaatδ^-^* mice, but not LPAATγ and LPAATα.

Lysophosphatidic acid acyltransferase δ is also highly expressed in white adipose tissue (WAT), particularly in epididymal and perirenal WAT (Prasad et al., 2011; Mardian et al., 2017). Male mice deficient in *Lpaatδ* gene have significant (by 40%) increase in the epididymal WAT weight with no effects on perirenal and inguinal WAT, as well as brown adipose tissue. The high PA and TAG levels in the epididymal WAT of *Lpaatδ^-^* mice is associated with an increase in adipocyte size rather than in adipocyte number. This is explained by an impaired lipolysis process due to reduced expression levels of adipose triglyceride lipase and phosphorylated hormone-sensitive lipase (Mardian et al., 2017). Here, a compensatory upregulation of LPAATα, LPAATβ, LPAATγ, and LPAATε occurs only in the perirenal WAT and not in the epididymal WAT. This adequate compensation mechanism is associated with normal tissue glycerolipid contents and, in turn, with normal tissue function (Mardian et al., 2017).

Interestingly, the loss of *Lpaatδ* gene pointed to the functional role of the specific pool of PA generated by LPAATδ enzyme. Indeed, although total PA level may be compensated in *Lpaatδ^-^* mice tissue by the induction of other LPAATs, the above studies indicated that the pool of PA generated by adaptive mechanisms is not able to functionally replace the LPAATδ-mediated production of PA and the downstream phospholipid derivatives that support specific cellular and tissue demands (as indeed shown in brain, soleus muscle and epididymal WAT in *Lpaatδ^-^* mice) (Bradley and Duncan, 2018; and references therein).

## Subcellular Localization of LPAATδ

According to Eto et al. (2014), murine LPAATδ localizes to the ER. Other authors reported that in mouse brain, LPAATδ resides on the MOM, but not on the MIM (Bradley et al., 2015). We recently demonstrated that both human and murine LPAATδ are targeted to both *trans-*Golgi membranes and mitochondria (Pagliuso et al., 2016).

In accordance with the endosymbiotic hypothesis, mitochondria of eukaryotes evolved from aerobic bacteria (Gray, 2012). As expected from this hypothesis, the MOM has similar composition to the plasma membrane and/or ER that may have surrounded symbiotic bacteria (Kuroda et al., 1998; and references therein), and it is not surprising that same or similar proteins are present in all these membranes (Kuroda et al., 1998; Colombo et al., 2005; Bhatt et al., 2008; Tamir et al., 2013; Marchi et al., 2014). We thus concluded that LPAATδ might localize both to the ER (from which it is transported to Golgi) and to the mitochondria (Pagliuso et al., 2016).

In addition to LPAATδ, there are other multipass transmembrane proteins that are targeted to both mitochondria and ER/Golgi. Mammalian diacylglycerol acyltransferase-2 (DGAT2) is a good example of such protein. Like LPAATδ, this enzyme is an acyltransferase, and reaction catalyzed by this enzyme, like LPAATδ-catalyzed reaction, belongs to the Kennedy pathway (Mcfie et al., 2011). DGAT2 contains two TMDs (Mcfie et al., 2014) and is localized to the ER, to the mitochondrial outer membrane, and to the lipid droplets (Mcfie et al., 2011). Mitochondrial targeting sequence contains few residues in the cytosolic portion of the protein, just upstream of the first TMD (Stone et al., 2009). This is a typical location of mitochondrial targeting signals (Rapaport, 2003; Stone et al., 2009). ER targeting signal resides within the first TMD (Mcfie et al., 2011), whereas lipid dropet targeting sequence is in the C-terminal cytosolic region of DGAT2 (Mcfie et al., 2018).

We carefully hypothesize that LPAATδ, like DGAT2, might contain separate targeting signals for mitochondria and for Golgi apparatus. The balance of LPAATδ amount between MOM and ER/Golgi membranes might be determined by comparative affinity of two signals for their respective organelles (Yogev and Pines, 2011). We expect that mutagenesis experiments will allow to specify the localization of these putative targeting signals. Possibly, as in the case of DGAT2, positively charged residues in the cytosolic portion of LPAATδ, just upstream of the putative second TMD, belong to the mitochondrial targeting sequence.

## Role of LPAATδ Enzyme in Membrane Fission of Golgi Membranes

Brefeldin A ADP-ribosylated substrate is the shorter splice isoform of CtBP1 protein, a member of the C-terminal binding protein (CtBP) family, known as CtBP1-S/BARS (Spano et al., 1999; Nardini et al., 2003; Valente et al., 2005; Corda et al., 2006). BARS (from here on) is a dual-function protein that in its dimeric NADH-bound conformation acts as transcriptional regulator in the nucleus, whereas in its p21-activated kinase 1 (PAK1)-phosphorylated monomeric conformation mediates membrane fission in the cystoplasm (Nardini et al., 2003, 2009; Yang et al., 2005; Colanzi et al., 2007, 2013; Liberali et al., 2008; Valente et al., 2013). BARS is a key member of a protein complex that is required for various membrane fission processes including basolaterally-directed post-Golgi carrier formation (Bonazzi et al., 2005; Valente et al., 2012), COPI-coated vesicle formation (Yang et al., 2005, 2008; Valente et al., 2012), macropinocytosis (Liberali et al., 2008; Valente et al., 2012), fluid-phase endocytosis and Golgi partitioning in mitosis (Hidalgo Carcedo et al., 2004; Bonazzi et al., 2005; Colanzi and Corda, 2007; Colanzi et al., 2007).

**FIGURE 4:**
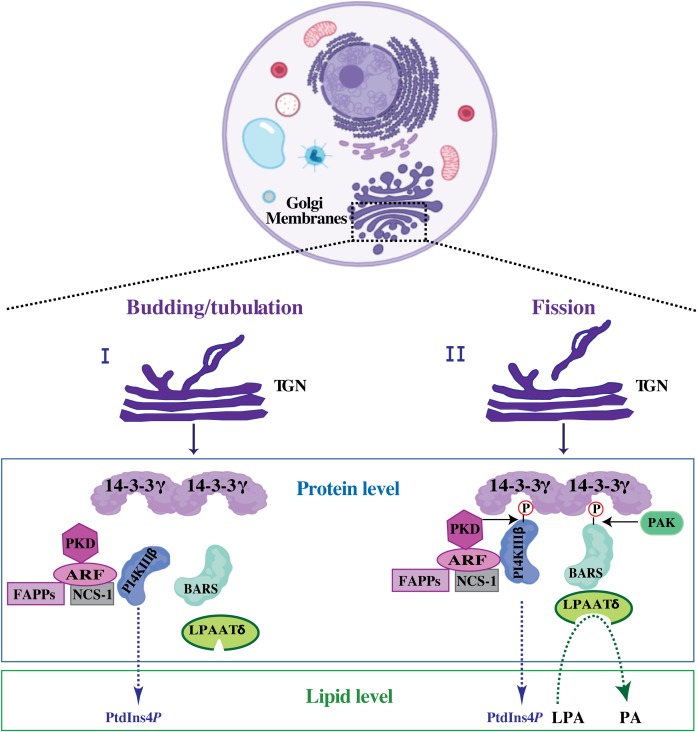
Putative mechanism of BARS-mediated membrane fission during post-Golgi carrier formation. Schematic representation of tubular carrier precursor that emerged out of specialized TGN-export domain during the budding/tubulation step (I), a process that precedes membrane fission (II). Upon fission event, the free post-Golgi carriers are released and directed toward the basolateral plasma membrane (usually with the employment of microtubular tracks and microtubule-based motors). At the protein level: upon activation ARF recruits PI4KIIIβ and PKD kinases onto the Golgi membranes and activates the PI4KIIIβ kinase leading to PtdIns*P*4 production. PI4KIIIβ kinase can be also activated by NCS-1, a well-known ARF interactor. ARF and the produced PtdIns*P*4-membrane pool promote the recruitment of FAPPs, which endorse membrane bending and budding/tubulation. PI4KIIIβ and BARS are then phosphorylated by PKD and PAK1, respectively (see text for details) allowing to the formation of a tripartite core complex where the phosphorylated kinase-active form of PI4KIIIβ is bridged to the phosphorylated fission-active form of BARS through 14-3-3γ dimer. Once incorporated into this complex, BARS binds to and activates LPAATδ enzyme leading to the local membrane conversion of LPA into PA. At the lipid level this enzymatic conversion of LPA into PA is central for the completion of the fission event. This figure is created using the web-based tool BioRender (https://biorender.com/library/).

We and others have previously shown that the BARS-induced fission on isolated Golgi membranes correlates with PA production starting from LPA and acyl-CoA and that this LPAAT catalytic reaction supports membrane fission (Weigert et al., 1999; Kooijman et al., 2003; Shemesh et al., 2003; Pagliuso et al., 2016). This LPAAT activity is associated with, rather than intrinsic to BARS (Gallop et al., 2005) as shown by the fact that: (i) the minimal BARS domain able to support COPI-coated vesicle fission does not incorporate this activity (Yang et al., 2005); and (ii) during purification of recombinant BARS from *E. coli*, bacterial LPAAT, known as PlsC, specifically binds BARS (Gallop et al., 2005; Pagliuso et al., 2016). These data prompted the search for evolutionary conserved interaction between BARS and the LPAAT enzymes from bacteria to mammals. We have shown that BARS, at the TGN, is incorporated in a well-defined protein complex (Valente et al., 2012), where it binds to and activates the LPAATδ enzyme catalyzing the production of a PA pool required to support membrane fission of the basolaterally-directed post-Golgi carriers (Pagliuso et al., 2016; Figure 4). Specifically, as the cargo protein reaches the TGN membranes, BARS upon PAK1-mediated phosphorylation at serine 147, that induces its monomeric fission-prone conformation, assembles in a complex where it binds to the 14-3-3γ adaptor (Valente et al., 2012, 2013). Through a 14-3-3γ dimer, BARS is in a tripartite core complex with the phosphoinositide kinase PI4KIIIβ and binds proteins implicated in post-Golgi carrier formation, such as ARF, NCS-1 (also known as frequenin) and PKD (Valente et al., 2012, 2013; Figure 4). This complex allows to spatially and temporally couple the budding/tubulation of post-Golgi carriers with their fission. The reversible and regulated formation of this complex enhances the efficiency of the fission machinery that assembles along the tubular carrier precursor emerging out of the TGN on the site where then fission will take place (Valente et al., 2012, 2013; Figure 4).

Recently we showed that, when incorporated into this complex, BARS binds to and activates LPAATδ and that this LPAATδ-mediated production of PA is required for fission of post-Golgi carriers (Pagliuso et al., 2016).

Many membrane fission processes require the presence of specific lipids. These lipids could be named lipid ligands (Gopaldass et al., 2017), or lipid factors (Danne et al., 2017), or lipid cofactors (Ramachandran, 2018). In some cases, fission might be energized via the energy used in the synthesis of lipid cofactors (Gopaldass et al., 2017). We claim that PA produced in the reaction catalyzed by LPAATδ is a lipid cofactor for BARS-mediated fission.

Lysophosphatidic acid acyltransferase δ localizes to the Golgi and to the MOM (Bradley et al., 2015; Pagliuso et al., 2016), see above. PA has been shown to play a role in mitochondrial dynamics by regulating membrane fusion and fission (Frohman, 2015; Kameoka et al., 2018; and references therein). We can hypothesize that in mitochondria, the role of a PA-producing enzyme as LPAATδ is also related, among other things, to membrane fission, similar to the role of LPAATδ at the Golgi complex. Such possible dual role of LPAATδ can be somewhat analogous to the function of hFis1 that is dual targeted to mitochondria and peroxisomes and regulates membrane fission of both organelles (Delille and Schrader, 2008).

Differently from BARS, another member of the CtBP family RIBEYE possesses acyltransferase activity (Schwarz et al., 2011). In mammals, CtBPs are encoded by two genes, CtBP1 and CtBP2. CtBP1 has two splicing variants, CtBP1-L and BARS (CtBP1-S/BARS), whereas CtBP2 has three splicing variants, CtBP2-L, CtBP2-S, and RIBEYE. RIBEYE contains a large N-terminal domain that is unrelated to the CtBPs (Maxeiner et al., 2016; Schwarz and Schmitz, 2017) and a C-terminal domain. This C-terminal domain is very similar to CtBP2 and BARS in the NAD(H)-binding domain and substrate-binding domain (ca. 88.5%; Kumar et al., 2002; Nardini et al., 2003) but with relatively different residues in the C-terminal region (ca. 50.6%; Kumar et al., 2002; Nardini et al., 2003). These differences in the C-terminal region could explain the intrinsic LPAAT activity owned by RIBEYE and not by BARS (Schwarz et al., 2011). Indeed, as reported, this is the region responsible for LPAAT activity and substrate binding (Schwarz et al., 2011). Moreover, this RIBEYE enzymatic activity was not due to contaminating proteins copurifying with RIBEYE (Schwarz et al., 2011). It has been proposed that PA generated by RIBEYE at synaptic ribbons facilitates synaptic vesicle trafficking (Schwarz et al., 2011). We hypothesize that in RIBEYE (UniProt accession number Q9EQH5-2), the N^792^H^793^xxxxD^798^ segment belongs to the LPAAT catalytic motif I, whereas E^701^GTR^704^ segment belongs to the LPAAT catalytic motif III and, perhaps, F^366^xxR^369^ pair belongs to the LPAAT catalytic motif II. However, we should consider that in LPAATs, the residue preceding D is almost always hydrophobic, see Supplementary Tables S1, S3, whereas in rat RIBEYE, this residue is hydrophilic N797. Moreover, the localization of two catalytic motifs in RIBEYE (motif III upstream of motif I) is very unusual, because in all (or almost all) other LPAATs, including LPAATδ, motif III is downstream of motif I (Korbes et al., 2016). Hence, most probably, LPAAT activity of RIBEYE evolved independently of the other LPAATs. We suppose that in RIBEYE dimer (whose crystal structure is not available yet), N^792^H^793^xxxxD^798^ motif of one protomer might be close to the E^701^GTR^704^ motif of another protomer. In the literature (Yamashita et al., 2007, 2014; Yuki et al., 2009; Eto et al., 2014; Pagliuso et al., 2016; and references therein), the catalytic activity-disrupting mutants of residues belonging to the catalytic motifs of LPAATs are reported, see above. Based on these data, we expect that in some of the mutants H793A, H793V, D798A, D798E, D798N, R369A, E701A, E701D, E701Q, G702L, R704A, R704K, the LPAAT activity of RIBEYE will be inhibited.

Following on the above considerations, we hypothesize that an evolutionary ancestor of BARS also possessed acyltransferase activity. BARS residues N232, H233 and D238 might form a vestige of an LPAAT catalytic motif I, whereas the E^141^GTR^144^ stretch might be a vestige of an LPAAT catalytic motif III. Possibly, an ancestor of BARS was simultaneously an LPAAT and a fission-inducing protein, it catalyzed production of PA and used this lipid as a cofactor in membrane fission reaction, similar to *Agrobacterium tumefaciens* PmtA that is an enzyme producing MMPE and a membrane fission-inducing protein that uses this lipid as a cofactor in fission reaction (Danne et al., 2017). Perhaps, later BARS gradually lost its LPAAT activity and simultaneously acquired an ability to bind and activate LPAATδ, and to use PA produced by LPAATδ as a cofactor in membrane fission reaction. Interestingly, the BARS residue R144 belonging to the putative vestigial catalytic motif E^141^GTR^144^ also belongs to the Rxx(pS) motif (pS is phosphorylated serine) that is involved in the interaction with 14-3-3γ adaptor protein needed for the formation of fully functional fission-inducing protein complex (Valente et al., 2012). Hypothetically, an ancestor of BARS gradually acquired the ability to bind 14-3-3γ, but in the course of this, the sequence motif E^141^GTR^144^, overlapping with the 14-3-3γ-binding site, lost the ability to be involved in LPAAT reaction. That is why, we hypothesize that simultaneously with acquiring the ability to bind 14-3-3γ, an ancestor of BARS gradually lost acyltransferase activity and acquired an ability to bind and activate LPAATδ.

## Conclusion

Lipid-modifying enzymes such as those of the LPAAT family discussed above are now recognized as central actors in diverse cell functions. Their central role is also testified by the neuronal and muscle pathologies, among others, that are linked to mutations in their structure. This is rather unexpected since the numerous isoforms of the different LPAATs could suggest that redundancy is there to protect the organism from the lack of a given membrane component, PA in this case.

We can assume that redundancy cures several defects, but still there are very specific functions in cell compartments or tissues that are finely controlled by the activity of a single member of the LPAAT family.

The challenge in this field is to build a complete picture of the lipid-modifying enzyme localization, function and regulation. This knowledge will help designing the approaches and tools that will allow complementing their activity; with these procedures, we may anticipate the treatment of diseases caused by defective lipid metabolism and/or altered membrane composition.

## Author Contributions

All authors wrote the manuscript. AF prepared the figures. MZ, CV and AF prepared the tables.

## Conflict of Interest Statement

The authors declare that the research was conducted in the absence of any commercial or financial relationships that could be construed as a potential conflict of interest.

## Footnotes

1 http://cctop.enzim.ttk.mta.hu

## Abbreviations

AGPAT: acylglycerophosphate acyltransferase
AMPA: α-amino-3-hydroxy-5-methyl-4-isoxazolepropionic acid
ARF: ADP ribosylation factor
BARS: brefeldin A ADP-ribosylated substrate
C14:0: myristic acid
C16:0: palmitic acid
C18:0: stearic acid
C18:1: oleic acid
C20:4: arachidonic acid
CGL: congenital generalized lipodystrophy
CL: cardiolipin
CoA: coenzyme A
DAG: diacylglycerol
DAGKs: diacylglycerol kinases
DHA: docosahexaenoyl acid
DHA-CoA: docosahexaenoyl-CoA
DHAP: dihydroxyacetone phosphate
DLCL: dilysocardiolipin
ER: endoplasmic reticulum
FAPP: four-phosphate-adaptor protein
G3P: glycerol 3-phosphate
GPAT: glycerophosphate acyltransferase
LPA: lysophosphatidic acid
LPAAT: lysophosphatidic acid acyltransferase
LPC: lysophosphatidylcholine
LPE: lysophosphatidylethanolamine
LPG: lysophosphatidylglycerol
LPI: lysophosphatidylinositol
LPS: lysophosphatidylserine
MIM: mitochondrial inner membrane
MLCL: monolysocardiolipin
MMPE: monomethyl-phosphatidylethanolamine
MOM: mitochondrial outer membrane
NCS-1: neuronal calcium sensor-1
NMDA: *N*-methyl D-aspartate
PA: phosphatidic acid
PAK1: p21-activated kinase
PC: phosphatidylcholine
PE: phosphatidylethanolamine
PI: phosphatidylinositol
PI4KIIIβ: phosphatidylinositol 4-kinase IIIβ
PKD: protein kinase D
PLA_2_s: phospholipases A2
PLD: phospholipase D
TAG: triacylglycerol
TGN: *trans*-Golgi network
TMD: transmembrane domain
*Tm*PlsC: *Thermotoga maritima* PlsC
WAT: white adipose tissue
